# Foam Rolling Intervention Improves Lactate Clearance After High-Intensity Exercise

**DOI:** 10.3390/sports12110303

**Published:** 2024-11-08

**Authors:** Kazuki Kasahara, Keita Oneyama, Takeru Ito, Masatoshi Nakamura, Genta Ochi

**Affiliations:** 1 Sanyudo Hospital, Yamagata 992-0025, Japan; 2 Department of Health and Sports, Niigata University of Health and Welfare, Niigata 950-3198, Japan; 3 Faculty of Rehabilitation Sciences, Nishi Kyushu University, Saga 842-8585, Japan; 4 Institute for Human Movement and Medical Sciences, Niigata University of Health and Welfare, Niigata 950-3198, Japan

**Keywords:** foam roller, lactate clearance, recovery, perceived exertion rating, cognitive function

## Abstract

The acute effects of a foam rolling intervention on lactate clearance and the impaired executive function associated with fatigue after high-intensity exercise remain unclear. This study examined whether foam rolling is an effective tool for fatigue recovery. Eighteen healthy adults without consistent exercise habits participated in this study. Participants performed high-intensity exercises, and the post-exercise foam rolling intervention was compared to the control condition. Measurements included lactate, vigor/fatigue by the Profile of Mood States 2nd Edition, cognitive function (cognitive task performance), leg and body rating of perceived exertion pre- and post-exercise, and post-intervention. Blood lactate concentrations post-foam rolling intervention (−7.3 ± 3.0 mmol/L) were significantly reduced among all participants. Increased lactate clearance by foam rolling correlated with a faster recovery of executive function for those with greater lactate clearance. However, cognitive fatigue was not observed after high-intensity exercise (*p* = 0.086, r = 0.41). Lactate clearance was not significantly correlated with the rating of perceived exertion in the foam rolling condition. The rating of the perceived exertion decreased with increased lactate clearance for those with greater lactate clearance in the control condition (leg: r = 0.778; body: r = 0.669). In conclusion, foam rolling intervention may be effective for exhausting exercise recovery.

## 1. Introduction

Many sports competitions involve high-intensity interval exercises such as repeated sprints and jumps, and athletes are required to perform these exercises for extended periods and with a limited recovery time between sessions or sets. High-intensity interval exercise, repeated with short rest periods, results in a noticeable decrease in performance (fatigue) in the second half of matches. Exercise-induced fatigue is thought to be caused by metabolic acidosis, muscle fatigue due to the depletion of energy sources [1], mental fatigue formation [2], and cognitive decline [3,4], all of which are thought to be related. Therefore, fatigue control and recovery methods are important for high performance in competitive sports that require high-intensity interval exercise.

Although peripheral blood lactate has long been considered a causative agent of metabolic acidosis, it is no longer considered as such. Rather, it is thought to reduce metabolic acidosis [5]. However, the increase in peripheral blood lactate coincides with the development of metabolic acidosis [6], which may be a valid indirect indicator of the development of muscle fatigue [7]. In addition, peripheral blood lactate is associated with cognitive decline [8] and may contribute to central functional decline. In this regard, rapid lactate removal after high-intensity exercise remains an important biomarker in improving peripheral and central fatigue.

Lactate clearance has been widely measured as a marker of post-exercise fatigue, and in recent years, recovery methods using foam rolling (FR) have received particular attention. The effects of no intervention (PAS) and FR conditions were compared in a previous study examining lactate clearance by FR [9]. The results showed a significantly greater lactate clearance in the FR condition than in the PAS condition after 30 min of intervention. The FR intervention could increase tissue blood flow [10,11] and decrease sympathetic excitatory stimulation with activation of the cutaneous receptors, thereby decreasing muscle tension [12,13]. As lactate removal is dependent on blood flow in the skeletal muscle [14], FR interventions may increase blood flow by applying mechanical pressure to muscles and other tissues, thereby promoting more efficient lactate removal. However, the immediate effects of FR on lactate clearance, which have been associated with reduced performance, are unknown. It remains unclear whether the lactate removal is due to the effects of FR, especially considering that the blood flow-promoting effects of FR may return to baseline 30 min post-intervention.

Therefore, this study aimed to investigate the immediate effects of a FR intervention on lactate clearance after high-intensity exercise using FR, which allows intervention in a small space within a limited time. We hypothesized that performing the FR intervention immediately after high-intensity exercise would improve lactate clearance more effectively than resting.

## 2. Materials and Methods

### 2.1. Experimental Approach to the Problem

A randomized repeated-measures experimental design was used to compare the two conditions, control and FR. Participants were instructed to visit the laboratory twice, with a ≥48 h break. Measurements were conducted before the exercise-intervention (pre), after the exercise-intervention (post), and 5 min after the end of the exercise-intervention (post 5 min). Between post and post 5 min, the FR intervention was performed in the FR condition, while participants in the control condition remained seated at rest. The completed psychological scales included the Profile of Mood States 2nd Edition (POMS2) [15] and participant rating of perceived exertion (RPE) [16]. The parameters measured were executive function, psychological scale scores, and blood lactate concentration.

### 2.2. Participants

A total of 22 right-handed Japanese-speaking young adults (12 males and 10 females) participated in this study conducted between October 2023 and January 2024. The inclusion criteria were no regular exercise habits; no daily use of a foam roller; no history of neurological, psychiatric, or respiratory diseases; and age between 18 and 30 years. Additionally, we confirmed that no participant had a history of cardiovascular disease or health-related concerns. All participants were instructed to refrain from eating or drinking anything other than mineral water 2 h before starting the experiment. A post hoc sensitivity analysis performed using this sample with 80% power and an α = 0.05 demonstrated sufficient sensitivity to detect repeated-measures effects exceeding f = 0.31, as computed using G*Power (3.1.9.2; The G*Power Team). Participants’ height and weight were measured before the experiment, followed by measurement of the circumference of both thighs. Thigh circumferences were measured to investigate the influence of the contact area between the FR and the thighs.

### 2.3. Procedures

#### 2.3.1. High-Intensity Exercise with a Progressive Load

High-intensity exercises with progressive loading were performed using an ergometer (828E; Monark, Sweden). Each participant performed an ergometer exercise for 3 min at a load of 0.5 KP as a warm-up, followed by a 1 min rest period. After the break, males and females performed ergometer exercises with a progressive increase in the load of 0.5 KP/min. Participants performed the ergometer exercises and were instructed to pedal on time using a metronome (Smart Metronome; Tomohiro Ihara, Japan) set at 60 bpm. The exercise was stopped when the pedaling speed could no longer be sustained. During the exercise, strong verbal encouragement was provided to elicit maximal effort. Exercise time and heart rate (HR) pre- and post-exercise-intervention (immediately before and after exercise) were measured during each session using an OH1 optical HR sensor (Polar, Finland) worn on the arm.

#### 2.3.2. FR Intervention

**Figure 1:**
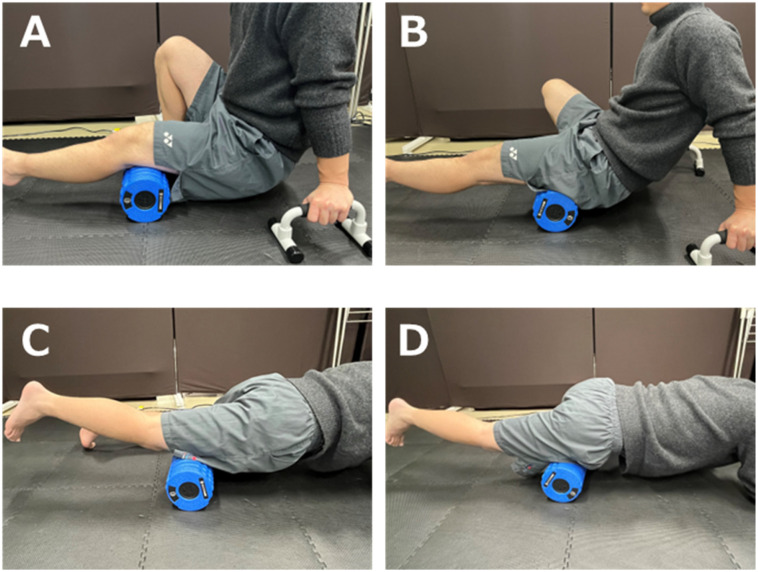
The FR intervention method using a foam roller. (**A**,**B**) The intervention method for knee flexors. A push-up bar was used, and the sole of the non-intervention side was placed in contact with the ground. (**A**) shows the distal part of the knee flexors, and (**B**) shows the proximal part. FR intervention was performed in the A-B range. (**C**,**D**) The intervention for the knee extensors, where the non-intervention knee was placed in contact with the ground. (**C**) Shows the distal part of the knee extensors and (**D**) shows the proximal part, and the FR intervention was performed in the range of (**C**,**D**). The FR intervention was performed bilaterally for 60 s at a speed of 1 repetition per 4 s in the range specified for each muscle (15 rolling in each intervention). FR, foam rolling.

Figure 1 shows the FR intervention method using a foam roller (Stretch Roll SR-002, Dream Factory, Umeda, Japan). A physical therapist instructed the participants prior to the intervention.

The FR intervention, performed on both the left and right sides, was initiated with knee flexors, followed by knee extensors. The FR area of the knee flexor muscle group was defined as the area proximal from the knee fossa to the sciatic tuberosity [17]. The knee extensor was defined as the superior anterior iliac spine at the top of the patella [18,19]. One FR cycle was defined as one distal rolling movement followed by one proximal rolling movement performed within 4 s [20]. The intervention intensity was defined as the maximum intensity that the participants could tolerate [18]. The intervention time for each area was 60 s, and the participants were instructed to prepare for the intervention for the next area during a 20 s interval. The FR intervention was performed for a total of 240 s, targeting the knee flexors and extensors of both legs. A metronome (Smart Metronome; Tomohiro Ihara, Japan) operating at 60 bpm was used for control. Additionally, during the FR intervention, both hands were used for support, ensuring that hand dominance did not affect the results.

#### 2.3.3. Psychological Measurements

POMS2 contains 35 items and evaluates seven mood states (anger-hostility, confusion-bewilderment, depression-ejection, fatigue-inertia, tension-anxiety, vigor-activity, and friendliness). This study used only a 10-item subset of vigor-activity and fatigue-inertia to reduce the psychological burden on the participants.

The RPE was recorded to assess the index as the subjective fatigue in the whole body and lower extremities at pre-, post- and post 5 min, using Borg’s scale, ranging from 6 to 20 [21].

#### 2.3.4. Blood Lactate Concentration

Blood lactate levels were measured at pre-, post-, and post 5 min. Lactate Pro 2 (AKRAY, Amstelveen, The Netherlands), a handheld point-of-care analyzer, was used for enzymatic amperometric detection. It requires 0.3 μL of a whole blood sample and a short time (15 s) to measure the lactate level. Lactate Pro 2 measurements range from 0.5 to 25.0 mmol/L. Therefore, only lactate values between 0.5 and 25, which fall within the normal range values of Lactate Pro 2, were included in this study. Lactate clearance was defined as the difference between lactate concentration immediately after exercise and the degree of recovery in lactate concentration after the FR or at post 5 min.

#### 2.3.5. Cognitive Task Performance

Schneider’s spatial Stroop task, which was created using web-building platforms (Lab.js v19.1.0), was used to measure executive function [22,23]. In each experiment, the participants made a spatially compatible key-press response to classify the spatial meaning of a stimulus presented at an irrelevant position. The stimulus meaning and position were either matched (congruent) or mismatched (incongruent) in the trial, with 16 congruent and 16 incongruent stimuli. Visual alert cues (squares) were briefly presented at two potential stimulus positions 500 ms before stimulus onset. Although previous studies used multiple stimulus types (arrows or words) and spatial dimensions (horizontal or vertical), this study employed arrow stimulus-type tasks. In addition, since preliminary experiments revealed that reaction times tended to be slower in vertical than in horizontal spatial dimension tasks, and the load on the task was assumed to be higher (fatigue was more likely to occur), vertical spatial dimension tasks were used in the present study.

**Figure 2:**
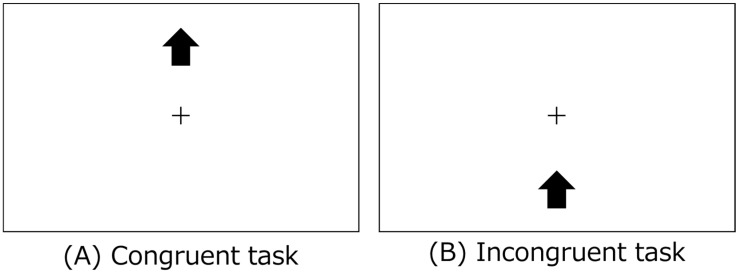
This is an example of Schneider’s spatial Stroop task. (**A**) In the Congruent task, the displayed position and the direction of the arrow match. (**B**) In the Incongruent task, the position displayed on the screen and the direction of the arrow are mismatched.

Participants pressed a key indicating up (“y”) or down (“b”) to classify the direction of an up- or down-pointing arrow positioned above or below fixation (Figure 2). The correct answer rates assigned to yes and no were 50%. Each stimulus was separated by an inter-stimulus interval showing a fixation cross for 2 s to avoid prediction of the timing of the subsequent trial. The stimulus remained on the screen until the patient responded or for 1 s. This study adopted Stroop interference, a specifically defined cognitive process, to elucidate the effects of an acute bout of exercise on executive function by calculating the incongruent–congruent contrast, which was assumed to represent Stroop interference.

Only data from 16 participants (eight males and eight females) were used for the cognitive task because data could not be obtained from two of the 18 participants.

### 2.4. Statistical Analyses

R software (4.3.2), Rstudio (2023.12.0 + 369), “EZR on R Commander” [24], and the “anovaun” package were used for data analyses (The R Foundation, Austria). A three-way analysis of variance (ANOVA) was performed to compare conditions, times, and sex. Mauchly’s sphericity test was used to determine whether sphericity was maintained. When this assumption was confirmed, a three-way ANOVA with Greenhouse–Geisser’s epsilon correction was performed; otherwise, only a three-way ANOVA was performed. A two-way ANOVA was performed to compare conditions and times. The effect size classification was set where η_p_^2^ < 0.01 was considered small; 0.02–0.1, medium; and >0.1, large [25]. A corresponding t-test with Holm’s correction was used to determine significant differences obtained from the ANOVA. Since normality was confirmed for Stroop interference, lactate, and RPE, Pearson’s correlation analyses were performed to clarify the relationships between the parameters and executive performance. Statistical significance was set a priori at *p* < 0.05.

## 3. Results

### 3.1. Patient Characteristics

Analyses were conducted with data from eighteen participants (ten males and eight females); four participants were excluded because of their inability to perform the FR intervention after high-intensity exercise. A post hoc sensitivity analysis was performed again and using this sample with 80% power and an α = 0.05; sufficient sensitivity was demonstrated to detect repeated-measures effects exceeding f = 0.35, as computed using GPower (3.1.9.2; The G*Power Team). Table 1 presents the participant characteristics.

### 3.2. Physiological Parameters

Table 2 presents the results of the HR, RPE, total exercise time, and kp-max in the control and FR conditions. No significant differences in the pre-test values were observed between the two conditions for any of the measures. HR increased significantly (*p* < 0.01) immediately post-exercise in both conditions (control: 101.9 ± 14.4, FR: 97.7 ± 22.5). RPE showed a significant main effect of time (lower extremities: F [1.54, 26.2] = 129.7, *p* < 0.001, η_p_^2^ = 0.884; body: F [2, 34] = 84.1, *p* < 0.001, η_p_^2^ = 0.8319). RPE (lower extremities/whole body) was significantly (*p* < 0.01) higher at post-exercise (lower extremities, control: 10.4 ± 3.0, FR: 10.6 ± 2.9; whole body, control: 9.1 ± 2.6, FR: 8.9 ± 3.5) and 5 min into the resting period (lower extremities, control: 6.7 ± 3.8, FR: 6.2 ± 3.1; whole body, control: 5.1 ± 3.9, FR: 4.7 ± 3.3) than at pre-exercise in both conditions but was significantly (*p* < 0.01) lower at 5 min into the resting period than at post-exercise (lower extremities, control: −3.7 ± 2.0, FR: −4.4 ± 2.4; whole body, control: −4.0 ± 2.3, FR: −4.2 ± 3.0). In addition, sex differences were observed in the total exercise time and post-exercise load, which were significantly (*p* < 0.01) higher in males than in females. However, no significant differences were observed between the two conditions.

**Figure 3:**
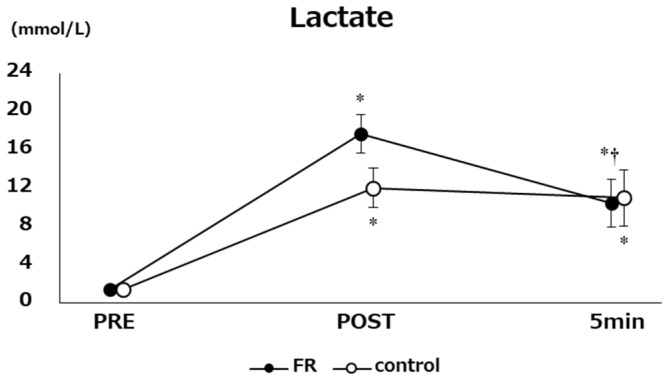
The changes in lactate at pre, post, and post 5 min are shown. *: significant difference with pre (*p* < 0.01); †: Significant difference with post (*p* < 0.01).

Figure 3 presents the changes in lactate levels. A three-way ANOVA (conditions vs. time vs. sex) showed no significant interactions (F [2, 32] = 1.00, *p* = 0.38, η_p_^2^ = 0.06). A two-way ANOVA (conditions vs. time) showed a significant interaction (F [2, 32] = 4.04, *p* < 0.05, η_p_^2^ = 0.20) and a main effect of time (F [2, 32] = 275.2, *p* < 0.0001, η_p_^2^ = 0.95). The post hoc test results in the control condition indicated significantly higher values at post-exercise (10.6 ± 2.0 mmol/L) and 5 min into the resting period (9.6 ± 2.9 mmol/L) than those at pre-exercise (*p* < 0.01). However, no significant difference was observed between post-exercise and 5 min into the resting period (−1.0 ± 2.1 mmol/L). In the FR condition, post-exercise (16.3 ± 2.0 mmol/L) and 5 min into the resting period (9.0 ± 2.5 mmol/L) values were significantly higher than at pre-exercise (*p* < 0.01). In addition, results at post 5 min (−7.3 ± 3.0 mmol/L) were significantly lower than those at post (*p* < 0.01).

### 3.3. Psychological Parameters

A three-way ANOVA (conditions vs. time vs. sex) showed no significant interaction between vigor (F [2, 32] = 1.69, *p* = 0.20, η_p_^2^ = 0.0953) and fatigue (F [2, 32] = 1.00, *p* = 0.38, η_p_^2^ = 0.059) in POMS2. A two-way ANOVA (conditions vs. time) showed a main effect of time on vigor (F [2, 32] = 7.5235, *p* < 0.01, η_p_^2^ = 0.32) and fatigue (F [2, 32] = 163.6, *p* < 0.0001, η_p_^2^ = 0.91). A post hoc test using Holm correction revealed that vigor was significantly lower in post-exercise (control: −2.2 ± 4.6, FR:−4.1 ± 5.4) than in pre-exercise conditions (*p* < 0.01). Fatigue was significantly higher at post (control: 12.7 ± 4.1, FR: 12.4 ± 3.7) and post 5 min (control: 8.1 ± 4.4, FR: 8.4 ± 4.8) than at pre-exercise (*p* < 0.0001). In addition, post-exercise values (control: −4.6 ± 2.9, FR: −4.0 ± 3.2) were significantly higher than those at post 5 min (*p* < 0.0001).

### 3.4. Relationship Between Parameters and Performance

**Figure 4:**
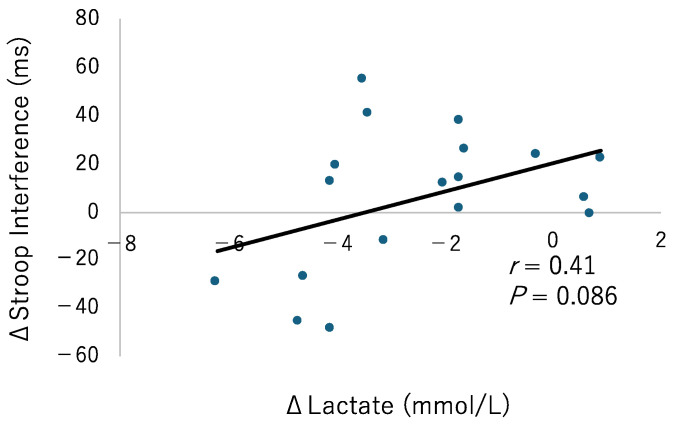
The correlation between the changes in lactate and Stroop interference in the FR condition are shown in the figure. The dotted lines indicate the regression lines for sexes, blue for males and orange for females.

Figure 4 displays the changes in lactate levels and executive function pre- and post-FR or rest intervention. Both lactate levels and executive function showed normality. A significant correlation was observed for the FR condition (r = 0.41, *p* = 0.086). Sex-specific results showed a significant correlation for males (r = 0.706, *p* < 0.05) but not for females (r = 0.13, *p* = 0.747). No significant correlation was found in the control group (r = −0.38, *p* = 0.118).

**Figure 5:**
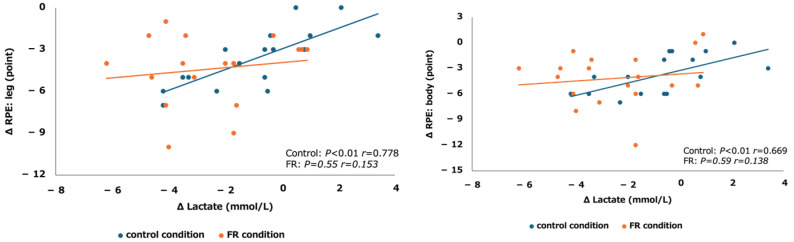
The correlations between the change in lactate and RPE lower extremities and whole body in FR and control conditions are shown separately.

Figure 5 presents the changes in lactate levels and RPE (lower extremities/whole body) before and after the intervention. No significant correlation was found under the FR condition; however, a significant correlation was found under the control condition (lower extremities: r = 0.778, *p* < 0.01; whole body: r = 0.669, *p* < 0.01).

## 4. Discussion

The acute effects of FR intervention on lactate clearance, psychological parameters, and cognitive function after a progressively increasing load exercise resulting in exhaustion, were examined to propose FR as a new recovery method that athletes can implement in a limited space. Our results revealed that the arterial blood lactate concentration, which increased after exhausting exercise, decreased in the FR condition regardless of sex, indicating that the FR intervention enhanced lactate clearance. Interestingly, cognitive function improved after the FR intervention, and this effect was significantly greater in males than in females. This finding indicates that FR intervention may be useful in improving exercise-induced cognitive decline, particularly in males.

This study showed significant increases in HR at pre- and post-exercise in both the FR and control conditions, without significant differences between them. Additionally, the Karvonen method [26] demonstrated that exercise intensity at post-exercise was 85.3 ± 11.0% in the FR condition and 83.6% ± 10.4% in the control condition, with no significant difference between the two conditions. Based on these results, we concluded that all participants performed the same amount of exercise under both conditions.

First, we examined the effects of FR intervention on lactate clearance. In the present study, ergometric exercise was performed with progressive loading by applying a strong load to the lower extremities, and FR intervention targeted the bilateral knee extensors and flexors. Arterial blood lactate concentrations increased significantly immediately after exercise under both conditions, and no difference was observed between the conditions. Thereafter, no lactate concentration changes were observed in the control condition. However, arterial blood lactate concentrations significantly decreased post-intervention in the FR condition. FR intervention immediately after exercise with fatigue was shown to be useful in increasing the lactate clearance, as no change in lactate clearance was observed in the control condition, in which 5 min of restful sitting was performed. A previous study reported that increased arterial blood lactate concentrations after high-intensity exercise were significantly lower in active recovery groups that performed low- or high-intensity exercises than in passive recovery groups that did not receive any intervention [27,28]. Our findings do not only align with those of previous research wherein FR interventions promote lactate clearance [9] but also present the first evidence that the lactate clearance effect can be observed immediately following a 4 min FR intervention. Although the physiological mechanisms remain unclear, the involvement of enhanced blood flow, due to the FR intervention, has been suggested [10,11,29]. In particular, given that lactate clearance is not promoted by mechanical stimuli such as massage therapy [30], it is possible that the direction of the FR intervention, aligned with the anatomical structure of the veins, plays a crucial role in facilitating lactate clearance.

Second, the effects of FR intervention on executive function were examined. In the present study, executive function task performance did not decrease before or after exercise until exhaustion, and progressive-load exercises leading to exhaustion did not induce cognitive fatigue. A previous study showed progressive exercise to exhaustion caused impaired cognitive function, including executive function [31]; however, its effects are not always clear. Whether laboratory training impairs executive function varies greatly from person to person and requires further testing. Exercise in extreme environments, such as hypoxic environments, decreases cognitive function [3,4]. To verify whether FR intervention is effective in restoring exercise-induced cognitive decline, it should be investigated under different exercise conditions, such as a hypoxic environment. This study demonstrated that the amount of lactate change was significantly correlated with changes in executive function task performance immediately after exercise and post-FR intervention in the FR condition. This result indicates that executive function may recover in patients with an increased lactate clearance. Previous studies have reported that elevated blood lactate levels after exhausting exercise are associated with significantly worsened attentional processes [8]. Therefore, an increased lactate clearance due to FR may contribute to improved cognitive underperformance due to increased lactate levels. Although no decline in executive function was observed in this study, it may be possible to propose a new effect of FR by testing it under experimental conditions in which a decline in executive function occurs.

We examined the effects of FR intervention on psychological parameters. The results indicated that FR intervention did not affect the vigor or fatigue of POMS2, supporting the findings of a previous study [32], in which FR intervention was administered to female basketball players and had no effect on perceived recovery (total quality recovery) or fatigue level. Additionally, regarding RPE, which represents perceived exertion, the results revealed a greater decrease in lactate clearance in the control condition, which was associated with a lower RPE. However, in the FR condition, no significant correlation was observed between the lactate clearance changes and RPE changes. These findings differ from those of previous studies, wherein a reduction in fatigue following the use of FR was noted [29,33,34]. These differences in results may be due to differences in the FR intervention methods. Pernigoni et al. [32] and Rey et al. [33] performed a 20 min FR intervention on the entire lower extremity, including the quadriceps, hamstrings, triceps, as well as abductor and adductor muscle groups. Conversely, this study found that the FR intervention lasted 4 min for knee extensors and flexors. A capacitance–response relationship has been found for the effect of FR on the range of motion increase [35]. The enhancement of psychological parameters through FR may also be associated with the capacity–response relationship, necessitating further research for a clearer understanding of that relationship. Furthermore, the participants’ exercise habits may also have influenced the results. While previous studies focused on individuals with regular physical activity habits [29,32,33,34], this study targeted participants without such habits. As a result, the FR intervention itself may have acted as a physical stressor, potentially limiting its positive effects on psychological measures. Therefore, although lactate clearance increased in the FR condition, the effect of improved perceived exertion might not have occurred.

This study has a few limitations. First, the mechanisms underlying these results remain unknown. The results indicated that FR intervention increased lactate clearance and concomitantly affected executive function. However, the detailed mechanism by which this occurs remains unclear. Future studies should examine the effects of FR intervention on regional and cerebral perfusion and changes in lactate clearance and cognitive function. Second, the target population was a study limitation. As aforementioned, FR intervention for male athletes showed fatigue recovery effects [33] but not for female basketball players [32]. Therefore, differences between those with exercise habits and older adults, as well as differences in athletic specialties and sex among athletes, should be examined in the future. In addition, the influence of prior knowledge about foam rollers was not examined. Although the participants in this study had no regular exercise habits and no prior experience using foam rollers, it is possible that their familiarity with foam rollers varied due to the widespread promotion of these devices for stretching and training. While the lack of significant differences in subjective exercise intensity between conditions suggests that the impact of prior knowledge was minimal, future research could clarify this influence by categorizing participants based on their prior knowledge and skills. Finally, the effect of FR intervention on exercise-induced cognitive decline (cognitive fatigue) was discussed. This study observed a correlation between increased lactate clearance and improved cognitive task performance; however, cognitive fatigue was not observed in the participants. Using exercise conditions, in which cognitive fatigue occurs, can assist in accumulating results to verify our findings.

## 5. Conclusions

The present study’s results revealed that the acute effect of FR intervention after exhausting exercise in healthy young adults was an increase in lactate clearance. Furthermore, the greater the effect, the greater the recovery of executive function. However, FR intervention did not decrease RPE after exhausting exercise. These findings suggest that the FR intervention may be a useful recovery method for promoting peripheral lactate clearance. However, athletes and coaches should carefully consider whether the recovery of physiological parameters or RPE should be prioritized when implementing FR interventions in the field. In addition to lactate clearance, further studies clarifying the FR intervention conditions for the recovery of cognitive function and fatigue, as well as identifying exercise conditions under which lactate clearance is improved and RPE does not improve, could potentially confirm FR as a new conditioning method for improving athlete performance.

## Figures and Tables

**Table 1:** Characteristics of participants (males: N = 10, females: N = 8).

Characteristics	Males (N = 10)	Females (N = 8)
Height (cm)	172.1 ± 5.0	158.4 ± 5.0 *
Weight (kg)	67.1 ± 8.4	48.1 ± 5.3 *
Age (years)	22.4 ± 1.3	21.0 ± 2.0
Thigh circumference (Right) (cm)	53.1 ± 4.9	46.7 ± 6.0 †
Thigh circumference (Left) (cm)	53.2 ± 4.3	47.1 ± 5.9 †

The following table shows the characteristics of the 18 participants (10 males and 8 females) in this study (mean ± standard deviation). *: Significant difference with males (*p* < 0.01), †: Significant difference with males (*p* < 0.05).

**Table 2:** Physiological parameters of each condition before (pre), immediately after exercise (post), and after intervention (post 5 min) are shown for each sex (mean ± standard deviation).

Conditions and Variables		Males	Females
Control			
	HR ^a^ pre (bpm)	76.9 ± 10.4	75.5 ± 6.4
	HR ^a^ post (bpm)	181.3 ± 11.9 *	174.4 ± 12.8 *
	RPE ^b^ pre: lower extremities/whole body (point)	6.4 ± 0.7/6.5 ± 0.7	7.9 ± 2.2/8.1 ± 2.3
	RPE ^b^ post: lower extremities/whole body (point)	18.3 ± 1.5 */16.8 ± 2.1 *	16.4 ± 1.8 */15.8 ± 1.6 *
	RPE ^b^ 5 min: lower extremities/whole body (point)	14.7 ± 2.9 *†/13.0 ± 3.4 *†	12.5 ± 2.2 *†/11.5 ± 2.7 *†
	total exercise time (sec)	651.0 ± 87.0	405 ± 92.5 **
	kp max (a.u.)	3.4 ± 0.4	2.3 ± 0.4 **
Foam rolling			
	HR ^a^ pre (bpm)	77.1 ± 12.2	81.6 ± 6.6
	HR ^a^ post (bpm)	181.6 ± 16.2 *	170.9 ± 25.2 *
	RPE ^b^ pre: lower extremities/whole body (point)	6.5 ± 1.0/6.6 ± 1.0	7.8 ± 2.2/8.4 ± 2.3
	RPE ^b^ post: lower extremities/whole body (point)	18.6 ± 1.4 */17.2 ± 2.6 *	16.5 ± 2.0 */15.1 ± 2.7 *
	RPE ^b^ 5 min: lower extremities/whole body (point)	14.4 ± 2.4 *†/12.7 ± 3.2 *†	11.9 ± 2.0 *†/11.4 ± 2.1 *†
	total exercise time (sec)	615.5 ± 138.5	396.6 ± 91.7 **
	kp max (a.u.)	3.2 ± 0.6	2.3 ± 0.4 **

^a^ HR: heart rate; ^b^ RPE: Rating of Perceived Exertion. *: Significant difference with pre (*p* < 0.01); †: Significant difference with post (*p* < 0.01); **: Significant difference with males (*p* < 0.01).

## Data Availability

All data generated or analyzed during this study are included in this article.
